# Exploring Attachment Dimensions in Individuals with Overweight or Obesity

**DOI:** 10.3390/bs15030305

**Published:** 2025-03-04

**Authors:** Silvia Tempia Valenta, Sara Ventura, Silvia Garelli, Valentina Vicennati, Massimiliano Beneventi, Alessandro Agostini, Uberto Pagotto, Nicola Filippini

**Affiliations:** 1 Department of Biomedical and Neuromotor Sciences, University of Bologna, 40126 Bologna, Italy; silvia.tempiavalenta@studio.unibo.it; 2 Department of Clinical and Surgical Sciences, University of Bologna, 40126 Bologna, Italy; sara.ventura7@unibo.it (S.V.); valentina.vicennati2@unibo.it (V.V.); beneventi_massimiliano@yahoo.it (M.B.); alessandro.agostin11@unibo.it (A.A.); uberto.pagotto@unibo.it (U.P.); 3 IRCCS Azienda Ospedaliero-Universitaria di Bologna, Policlinico di Sant’Orsola, 40126 Bologna, Italy; silvia.garelli@aosp.bo.it; 4 IRCCS San Camillo Hospital, 30126 Venice, Italy

**Keywords:** attachment, obesity, overweight, binge eating, quality of life

## Abstract

**Introduction.** Obesity is a complex condition associated not only with physical health risks but also with social discrimination and a reduced quality of life (QoL). Psychological factors, including attachment styles, may play a role in emotional regulation and eating behaviors. According to attachment theory, insecure attachment is linked to difficulties in managing emotions and an increased likelihood of engaging in dysfunctional eating patterns. This study aimed to investigate the relationship between past and present attachment styles, binge-eating behaviors, and QoL scores in individuals with overweight or obesity (BMI ≥ 25 kg/m^2^) compared to those with a BMI in the normal weight range (18.5–24.9 kg/m^2^). **Methods.** A cross-sectional study evaluated 96 women with overweight/obesity and 96 women with normal weight. Psychological measures included the Parental Bonding Instrument (PBI), the Attachment Style Questionnaire (ASQ), the Binge Eating Scale (BES), and the Obesity-Related Well-being questionnaire (ORWELL 97). Data analysis included between-group comparisons of attachment dimensions and hierarchical regression to examine associations with the QoL score. **Results.** Participants with overweight/obesity showed less secure attachment (U = 5508, *p* = 0.019) and more avoidant attachment styles (U = 3825, *p* = 0.042) compared to participants with normal weight. Conversely, no significant differences were observed in PBI scores. Regression analysis revealed that attachment anxiety (B = 0.83, *p* = 0.009) and binge-eating behaviors (B = 0.57, *p* = 0.003) were significantly associated with a lower QoL. **Conclusions.** Obesity is associated with avoidant attachment in adulthood, but no differences were found in parental attachment when compared to individuals with normal body weight. Anxious attachment in individuals with obesity is strongly linked to reduced QoL, underlying the role played by attachment-related factors in the psychosocial challenges individuals often have to face.

## 1. Introduction

One of the earliest scientific manuscripts concerning obesity, dated 1851, described it as an incurable and invariably fatal condition, associated with malformations and intellectual disabilities (Chambers, 1851). Despite the tremendous advances made by medicine and society over the past century and particularly in recent decades, prejudices and discrimination related to obesity persist in subtle but impactful ways (Rubino et al., 2020). Some studies have shown that certain health risks associated with obesity are comparable in magnitude to those linked to being underweight (Thompson et al., 1999; Roh et al., 2014). However, cultural norms and media portrayals often idealize extreme thinness, despite numerous awareness campaigns advocating for body diversity and inclusion (Polivy et al., 1986; Dilling & Petersen, 2022). As an indirect result, individuals with obesity and/or binge-eating behaviors may experience discrimination and social exclusion, which can negatively impact their attachment styles, social relationships and overall quality of life (Sikorski et al., 2011; Puhl & King, 2013).

Attachment is a biologically-based behavioral system derived from evolution that regulates behaviors and feelings within the context of interpersonal relationships (Bowlby, 1977; Goldberg, 2014). Research on attachment has shown that early and repeated interactions between infants and caregivers determine the development of implicit cognitive schemas (working internal models, WIMs, in the terminology of attachment theory) that guide infant behaviors when developing their relationships (Goldberg, 2014). WIMs determine an individual’s attachment style, or their mode of relating to others, a pattern developed in childhood that generally persists throughout life (Levy et al., 2011). If interactions between infants and caregivers are characterized by a loving presence, mirroring and understanding of needs, and appropriate responses to those needs, the child may develop a secure attachment style. In adulthood, an individual with secure attachment has a positive representation of both the self and others (Bartholomew et al., 2001). They exhibit good relational abilities and, using strong mentalization skills, they are capable of seeking and receiving support in times of adversity (Keller, 2013). Conversely, if caregiving is characterized by inconsistency, distance, unreachability, or unavailability, the child may develop an insecure attachment style, marked by attachment anxiety or attachment avoidance (Bowlby, 1978). Avoidant attachment is thought to result from early interactions characterized by inconsistency, intrusiveness, and disturbance (Edelstein & Shaver, 2004). In this case, adults tend to develop a positive self-representation but a negative representation of others, leading to avoidance of closeness and intimacy (Li & Chan, 2012). In adversity, the avoidant individuals attempt to solve problems autonomously while rejecting support from others (Li & Fung, 2014). On the other hand, an individual with anxious attachment has a negative representation of the self, seeing themselves as unlovable, while actively seeking closeness with others they perceive as unavailable but essential (Li & Chan, 2012). In adversity, individuals with anxious attachment seek closeness with others and tend to amplify signals of discomfort and distress (Campbell & Marshall, 2011).

Research based on attachment theory in the context of obesity has highlighted the presence of an insecure attachment style and hypothesized a bidirectional relationship between insecure attachment and obesity (Anderson & Whitaker, 2011). On the one hand, the presence of obesity can pose a barrier to interpersonal relationships in light of aspects of discrimination and social discrimination, fostering a negative view of the self or others, thus leading to a “shift” in attachment style toward insecurity (Alexander, 2017; Cassin et al., 2018). On the other hand, insecure attachment may promote the development of dysfunctional eating behaviors as a possible form of comfort and reward (Hardman et al., 2016; Mamo & Louka, 2022).

In this study, we aimed to (1) explore the past and present attachment styles in a group of individuals with obesity or overweight seeking treatment, compared to a group of individuals with a body weight within the normal range and to (2) examine the relationship between dimensions of attachment and the presence of binge-eating symptoms with quality of life (QoL) as it relates to living with obesity. We hypothesized that there are higher levels of insecure attachment among individuals with obesity or overweight and that there is an association between insecure attachment and reduced quality of life.

## 2. Materials and Methods

### 2.1. Study Design and Participants

A cross-sectional study was conducted to assess psychological and behavioral variables in a sample of 96 women with overweight or obesity. The assessment was carried out to evaluate the attachment style, the presence of binge-eating symptoms, and the quality of life (QoL). Participants were recruited from among individuals seeking support for weight management or health conditions related to excess weight at the Division of Endocrinology, St. Orsola-Malpighi Hospital, Bologna, Italy. The inclusion criteria for participation in the overweight or obesity group were: (1) a body mass index (BMI) ≥ 25 kg/m^2^, (2) self-identifying as female, (3) being 18 years of age or older, and (4) being a native Italian speaker. Exclusion criteria included having incomplete assessments, being diagnosed with hormonal disorders, ongoing psychotic disorders, cognitive impairment or intellective disability.

A control group of 96 women with body weight within the normal range (18.5–24.9 kg/m^2^), good overall health, and living in the same geographic area as the patients’ group was randomly chosen from the general population. Inclusion criteria for the control group were: (1) a BMI within the normal range, (2) self-identifying as female, (3) being 18 years of age or older, and (4) being a native Italian speaker. Exclusion criteria included having incomplete assessments, a diagnosis of chronic medical conditions, ongoing psychotic disorders, cognitive impairment, intellectual disability, or long-term medication use.

The decision to include only women was based on the need to control for potential gender-related differences in psychological dimensions, attachment styles, and eating behaviors. Research suggests that men and women may differ in their experiences of attachment, emotional regulation, and disordered eating patterns, which could introduce additional variability into the results (Gorrese & Ruggieri, 2012). By focusing solely on women, this study aimed to maintain a more homogeneous sample, reducing the influence of gender as a confounding factor.

Psychometric questionnaires were administered by trained psychologists from the Department of Medical and Surgical Sciences (DIMEC). This study was approved by the local Ethics Committee (protocol code 149/2015/U/Sper) and performed in accordance with the ethical principles of the Declaration of Helsinki and Good Clinical Practice guidelines. All participants were fully informed about the purpose of the study and provided their voluntary informed consent to participate.

### 2.2. Procedures and Psychometric Measures

Socio-demographic data, including age, gender, education, anthropometric data (i.e., BMI and waist circumference), presence of metabolic-related comorbidities (i.e., hypertension, dyslipidemia, and diabetes type 2), and use of medications, were collected during the recruitment stage for the group with obesity or overweight. Additionally, data collected for this group included the Parental Bonding Instrument (PBI) (Parker et al., 1979), the Attachment Style Questionnaire (ASQ) (Feeney et al., 2014), the Binge Eating Scale (BES) (Timmerman, 1999), and the Obesity-Related Well-being Questionnaire (ORWELL 97) (Mannucci et al., 1999).

The control group was included solely for comparisons on the attachment style. For this group, data on the PBI and the ASQ tests were collected. This group was designed to exclude individuals with psychological disorders, including eating disorders, to ensure a focused and unbiased comparison of attachment styles. As a result, questionnaires specifically related to eating disorders and obesity, such as the BES and the ORWELL 97, were not administered.

#### 2.2.1. Parental Bonding Instrument (PBI)

The PBI is a 25-item self-report questionnaire that evaluates perceived parental styles during the participant’s first 16 years of life (Parker et al., 1979; Scinto et al., 1999). The PBI includes two subscales: (1) care (12 items), which measures parental behaviors related to affection, sensitivity, and accessibility; and (2) overprotection (13 items), which measures attitudes reflecting control, interference, and encouragement of autonomy or independence. Participants rate each item for both parents on a four-point scale. The PBI has demonstrated reliability and validity, with results being independent of the parent’s gender (Parker et al., 1979). Data on parental bonding were collected for both the overweight/obesity group and the control group. For this study, the validated Italian version of the PBU was used (Scinto et al., 1999).

#### 2.2.2. Attachment Style Questionnaire (ASQ)

The attachment style was assessed using the ASQ, a 40-item self-report questionnaire designed to evaluate attachment dimensions (Pedrazza & Boccato, 2010; Feeney et al., 2014). The ASQ includes a series of statements such as “I think it is important that people can rely on each other,” and participants were asked to rate their level of agreement or disagreement on a 6-point Likert scale, where 1 = “totally disagree” and 6 = “totally agree.” The ASQ contains five subscales: (1) confidence (8 items), describing secure attachment; (2) discomfort with closeness (10 items); and (3) relationships as secondary (7 items), assessing attachment avoidance; as well as (4) need for approval (7 items); and (5) preoccupation with relationships (8 items), describing attachment anxiety (Feeney et al., 2014). Attachment data were collected for both the overweight/obesity group and the control group. For this study, the validated Italian version of the ASQ was used (Pedrazza & Boccato, 2010).

#### 2.2.3. Binge Eating Scale (BES)

For the group with obesity or overweight, eating behavior was assessed using the BES, a 16-item self-report questionnaire that evaluates behavioral, cognitive, and emotional signs of binge eating (Timmerman, 1999; Imperatori et al., 2016). Each item presents statements, and participants choose from among three or four responses of increasing severity. BES is not a diagnostic tool but rather a measure of binge-eating symptoms. Total scores range from 0 to 46, with scores below 18 indicating no significant binge eating, scores between 18 and 26 suggesting binge-eating behaviors, and scores above 27 indicating clinically significant binge eating. For this study, the validated Italian version of the BES was used (Timmerman, 1999; Imperatori et al., 2016).

#### 2.2.4. Obesity-Related Well-Being Questionnaire (ORWELL 97)

Quality of life in individuals with obesity or overweight was assessed using the ORWELL 97, an Italian 18-item self-report questionnaire that evaluates symptoms and functional limitations associated with obesity, as well as their impact on the patient’s subjective well-being (Mannucci et al., 1999). The ORWELL 97 comprises three subscales: (1) symptoms (5 items), measuring obesity-related somatic symptoms and physical functioning; (2) discomfort (7 items), evaluating the emotional impact of obesity and related worries; and (3) impact (6 items), assessing the effects of obesity on family relationships, role functioning, and social networks. Higher scores indicate greater body uneasiness and lower quality of life. Data on binge-eating behavior and quality of life were not collected for the control group.

### 2.3. Statistical Analyses

Data analysis was carried out using the IBM Statistical Package for the Social Sciences (SPSS) Statistics software (Version 24.0). Statistical significance was set at *p* < 0.05. A priori power analysis indicated that a minimum of 64 participants per group was required to detect moderate effects in between-group comparisons (Mann-Whitney U test), while the current sample of 192 participants was sufficient for hierarchical regression analyses examining small-to-moderate effect sizes.

Categorical variables were analyzed by determining frequencies (n) and percentages (%). Continuous variables were assessed for skewness, kurtosis, and adherence to a normal distribution using the Shapiro–Wilk test. For variables following a normal distribution, the mean and standard deviation (SD) were calculated to summarize the central tendency and variability. For variables that did not adhere to a normal distribution, the median was used as the measure of central tendency, and the mean rank was included to provide additional context for group comparisons. A distribution comparison (i.e., Mann–Whitney) was conducted to determine whether there was a significant difference in age distribution between the two groups (obesity vs. normal weight).

Group comparisons of attachment dimension scores (overweight/obesity vs. normal weight) were analyzed using the Mann–Whitney test. Hierarchical multiple regression analyses were conducted to examine the association between psychological dimensions (PBI, ASQ, BES) and quality of life (QoL). In the first step, only psychological dimensions were included to assess their independent contribution to QoL. In the second step, demographic and anthropometric variables (e.g., age and BMI) and clinical variables (e.g., presence of metabolic-related comorbidities) were added as covariates to evaluate their additional impact on QoL. Throughout the analysis, QoL served as the dependent variable. Collinearity diagnostics confirmed that PBI, ASQ, BES, and QoL met acceptable thresholds (VIF < 5, Tolerance > 0.2), ensuring reliable interpretation of the regression results.

## 3. Results

### 3.1. Sample Sociodemographic and Clinical Characteristics

For detailed information please refer to Table 1 and the Supplementary Materials. The study included 96 female individuals with overweight or obesity, with a mean age of 49.3 ± 12.3 years, a mean BMI of 33.4 ± 3.7 kg/m^2^, and an average waist circumference of 103.9 ± 10.4 cm. Most participants were married, whilst a smaller proportion was in a committed relationship or widowed. Regarding occupation, more than one third of the sample reported full-time employment, with others distributed across homemakers, retirees, and other categories. In terms of menopause status, almost two-thirds of participants were not in menopause, while nearly one-third were in natural menopause. Among clinical conditions, hypertension was the most prevalent, followed by diabetes type 2 and dyslipidemia, all of which were under active medical treatment. The control group included 96 women with a mean age of 49.2 ± 10.7 and BMI within the normal range. No difference was observed for age between the two study groups (overweight/obesity vs. control) (U = 4373.500, *p* = 0.466).

### 3.2. Comparison of Attachment Dimensions Between Individuals with Overweight/Obesity and Control Group

For detailed information please refer to Table 2. The comparison of attachment dimensions between individuals with overweight/obesity and those with a body weight within the normal range revealed significant differences on two subscales of the ASQ. Specifically, individuals with overweight/obesity had significantly lower scores on the confidence subscale and higher scores on the discomfort with closeness subscale compared to those with a normal body weight. No significant differences were observed between the two groups on the other ASQ subscales or any of the PBI subscales. 

### 3.3. Factors Associated with Quality of Life (QoL)

For detailed information please refer to Table 3. In the first stage of hierarchical regression analysis, psychological dimensions (PBI, ASQ, BES) were entered into the model to examine their association with QoL. The results revealed that these psychological factors explained 23.6 percent of the variance in QoL (adjusted R^2^ = 0.236), indicating a moderate contribution to overall well-being. Among the different psychological dimensions, ASQ concern for relationships emerged as a statistically significant factor, showing that individuals with higher levels of attachment-related anxiety experienced lower QoL. Similarly, the total BES score was significantly associated with lower QoL.

In the second stage, demographic and anthropometric variables (age, BMI) and clinical variables (hypertension, diabetes type 2, dyslipidemia) were added as covariates. The inclusion of these variables improved the explanatory power of the model, increasing the adjusted R^2^ to 0.311, indicating that an additional 7.5% of the variance in QoL was accounted for by these factors. Among the clinical variables, hypertension and diabetes type 2 were found to be significantly associated with lower QoL. Despite the addition of the covariates, the psychological dimensions remained significant contributors to QoL. The ASQ preoccupation with relationships and the BES total score maintained their statistically significant association with lower QoL scores. Furthermore, ASQ confidence emerged as significant, indicating that individuals with higher confidence in relationships experienced better QoL. 

## 4. Discussion

The present study analyzed attachment dimensions and factors associated with the QoL of 96 female individuals with overweight or obesity. Attachment dimensions for the overweight/obesity group were compared to individuals of normal body weight. Significant differences were found on two subscales of the ASQ: overweight/obese individuals scored lower on confidence (U = 5508, *p* = 0.019) and higher on discomfort with closeness (U = 3825, *p* = 0.042). No significant differences were found between the two groups on any of the PBI subscales assessing parental bonding. Hierarchical regression revealed that psychological dimensions explained 23.6% of the variance in QoL (ORWELL 97), with ASQ preoccupation with relationships (B = 0.83, *p* = 0.009) and BES total score (B = 0.57, *p* = 0.003) showing significant associations with a lower QoL. After adding demographic, anthropometric, and clinical covariates, the model explained 31.1% of the variance, with hypertension and diabetes type 2 significantly associated with a lower QoL. Psychological dimensions continued to contribute significantly, with ASQ preoccupation with relationships, BES total score, and ASQ confidence showing significant associations with QoL.

Research based on attachment theory has shown an insecure attachment style among individuals with obesity (Anderson & Whitaker, 2011; Diener et al., 2016). Insecure attachment can manifest as either anxious attachment (characterized by high attachment anxiety and a need for approval) or avoidant attachment (characterized by emotional detachment and difficulty forming close relationships) (Sroufe et al., 1983; Bartholomew et al., 2001; Schreiber et al., 2021). Previous literature in this field has shown that people with obesity tend to show insecure attachment on both the anxious (Mills, 1994; Nancarrow et al., 2018; Wilkinson et al., 2018) and avoidant sides (Kay, 1981; Glucksman, 1989; Mills, 1994; Aarts et al., 2014). Our results support these findings. Indeed, we showed that individuals with overweight/obesity had higher scores on discomfort with closeness, indicative of a greater tendency toward avoidant attachment. Regarding avoidant attachment, previous research has hypothesized that individuals with this attachment style may use food as a coping mechanism to regulate emotions, avoiding reliance on interpersonal relationships and that a detachment from emotional intimacy may hinder the ability to seek support in stress management, potentially exacerbating unhealthy eating behaviors and weight gain (Monteleone et al., 2017; Faber et al., 2018). Furthermore, avoidant attachment has been associated with negative body image, as individuals may repress body-related emotions or avoid self-reflection, leading to maladaptive health behaviors such as disordered eating patterns (e.g., restrictive dieting or emotional overeating), reduced physical activity, neglect of medical care, and increased engagement in unhealthy coping mechanisms like substance use or social withdrawal (Calvo et al., 2022). On the other hand, it cannot be excluded that the presence of overweight and obesity may promote a negative vision of the self and psychological defense mechanisms based on avoidant attachment behaviors such as isolation and discomfort with intimacy.

Our study explored both present attachment styles using the ASQ, and childhood parental bonding using the PBI. We found no significant differences in PBI dimensions, such as need for approval, concern for relationships, or overprotective parenting, between individuals with overweight/obesity and those with a body weight in the normal range. Conversely, we found differences in the ASQ scores, reporting reduced confidence and increased discomfort with closeness in the group with overweight/obesity relative to the normal weight group. Taken together, these results suggest that the adult attachment style may undergo changes, potentially due to chronic individual or social challenges associated with obesity. Attachment style is known to evolve over the course of life, especially in response to adverse experiences (Davila et al., 1997; Zhang & Labouvie-Vief, 2004; Yilmaz et al., 2022; Cushing et al., 2024). Our findings suggest a potential bidirectional relationship between attachment insecurity and eating behaviors, where insecure attachment may contribute to the use of food as a means of emotional regulation, increasing the risk of weight gain. Conversely, the psychological and social challenges associated with living with overweight or obesity may reinforce or exacerbate attachment insecurity over time. This pattern aligns with mechanisms observed in other chronic conditions (Agostini et al., 2016), where sustained emotional distress and maladaptive coping strategies can lead to shifts in attachment styles. It is possible that, rather than a static characteristic, attachment insecurity evolves in response to prolonged psychosocial stressors, including experiences of weight-related bias and social exclusion. Future research should explore how stigma, discrimination, and other psychosocial factors shape this dynamic relationship, potentially contributing to a self-reinforcing cycle that impacts both psychological well-being and long-term weight management.

Analyses involving the QoL score have shown an association between binge-eating behaviors (BES) and lower QoL, consistent with a large body of previous literature. Most notably, we found a significant association between anxious attachment and a reduced QoL score, but no association between avoidant attachment and a reduced QoL. Since previous research has shown that both insecure attachment styles are associated with reduced well-being (Ponizovsky & Drannikov, 2013; Sechi et al., 2020; Darban et al., 2020), this finding aligns with a psychological and social interpretation of the phenomenon. An avoidant attachment style may offer some “protective factors”, as individuals with this style tend to rely on self-reliance and avoid social interactions that might expose them to prejudices or discrimination (Cassidy, 1994; Mikulincer & Shaver, 2019). In contrast, individuals with anxious attachment may experience greater distress in relational difficulties, amplifying the negative impact on QoL (Campbell & Marshall, 2011). Our results support previous findings showing that continued attachment anxiety may lead to relational issues that may in turn intensify negative thinking, over-activate the attachment behavioral system, and ultimately lead to a reduction in well-being (Myers & Wells, 2015; Akkuş & Yılmaz, 2021). In addition, when considering the weight-related discrimination that this demographic often faces in their interpersonal relationships, attachment anxiety may further amplify the impact of obesity on emotional difficulties and QoL. Globally, these findings may suggest that anxious attachment can exacerbate the challenges of living with obesity in a stigmatizing social environment, whereas avoidant attachment may serve as a defensive mechanism carried out to mitigate psychosocial burdens.

The limitations of this study included its small sample size and cross-sectional design, which prevented the establishment of a clear directionality in the observed associations. Another limitation was the inclusion of only women, which, although intended to reduce gender-related biases, restricted the generalizability of the findings to men and non-binary individuals. Additionally, recruiting participants from a hospital setting, where individuals were already seeking help, introduced a potential selection bias that may not have fully represented the broader population with overweight or obesity. Other relevant variables, such as socioeconomic characteristics, personality traits, and mental health status, were not assessed, which may have influenced the findings. Furthermore, participants with overweight and obesity were not stratified based on BMI scores to maintain an even sample size across the two groups. While this approach ensured comparability, it may have overlooked potential differences in attachment styles, binge-eating behaviors, or quality of life across different severity levels of overweight and obesity. Moreover, the control group with normal body weight had data only on attachment (ASQ/PBI), limiting comparisons on other psychological and clinical measures. Future research should address these limitations by including a larger and more stratified sample, adopting a longitudinal design and examining a broader range of sociodemographic and clinical factors. Additionally, future studies should further explore the impact of stigma and discrimination, as these factors are known to influence eating behaviors and related symptoms (Rodrigues et al., 2022; Tempia Valenta et al., 2024).

In conclusion, the present results provide a first hint about the association between overweight/obesity and avoidant attachment in adulthood, an aspect that seems to reflect a tendency toward emotional detachment and discomfort with closeness. Conversely, we did not observe any significant difference in parental attachment compared to individuals with normal body weight, suggesting that early childhood attachment patterns may not differ significantly between groups but may change later in life. This finding highlights the potential influence of chronic challenges associated with a body weight that does not conform to Western standards. Secondly, the present study shows how anxious attachment and binge-eating behaviors are strongly associated with poorer QoL in individuals with overweight/obesity. This could potentially imply that avoidant attachment may serve as a partial coping mechanism by promoting self-sufficiency. These findings emphasize the importance of considering attachment-related factors in clinical practice and addressing relational difficulties to improve emotional well-being and QoL in this population, as well as the importance of social policies that promote body positivity to alleviate social pressures that can impact and attachment styles and overall well-being.

## Figures and Tables

**Table 1:** Sociodemographic and clinical characteristics of the overweight/obesity group (n = 96).

Variable		Mean ± SD
**Age (in years)**		49.3 ± 12.3
**Children**		1.1 ± 0.8
**Pregnancies**		1.3 ± 0.8
**Body mass index (kg/m^2^)**		33.4 ± 3.7
**Waist circumference (cm)**		103.9 ± 10.4
**Variable**	**Answer**	**n, %**
**Menopause status**	Not in menopause Natural menopause Surgical menopause	60 (62.5%) 31 (32.3%) 5 (5.2%)
**Hypertension, yes/no**		46 (47.9%)/50 (52%)
**Dyslipidemia, yes/no**		68 (70.8%)/28 (29.1%)
**Diabetes type 2, yes/no**		61 (63.5%)/35 (36.4%)

**Table 2:** Comparison of attachment dimensions between individuals with overweight/obesity and individuals with body weight within the normal range using the Mann–Whitney U Test.

		Individuals with Overweight/Obesity (N = 96)	Individuals with Normal Body Weight (N = 96)	Mann–Whitney U Test	
Scale	Subscale	Median (Mean Rank)	Median (Mean Rank)	U	*p*
**PBI**	Care (mother)	24 (94.8)	27 (97.2)	4677	0.759
	Overprotection (mother)	16 (99.3)	14 (92.8)	4251	0.418
	Care (father)	25 (97.5)	23 (93.6)	4325	0.621
	Overprotection (father)	14.5 (98.2)	13.5 (92.9)	4261	0.507
**ASQ**	Confidence	33 (87.1)	34 (105.9)	5508	0.019 *
	Discomfort with closeness	35.5 (104.7)	34 (88.34)	3825	0.042 *
	Relationships as secondary	16.5 (102.0)	15 (91.1)	4085	0.174
	Need for approval	21 (100.8)	20 (92.2)	4195.5	0.283
	Preoccupation with relationships	27 (96.8)	27 (96.2)	4576.5	0.935
**BES**	Total score	11	-		-
**ORWELL 97**	Total score	51	-		-

*Legend.* PBI, Parental Bonding Instrument; ASQ, Attachment Style Questionnaire; BES, Binge Eating Scale; ORWELL 97, Obesity-Related Well-being Questionnaire. *Notes. p* < 0.05 (*).

**Table 3:** Hierarchical regression analysis examining factors associated with quality of life (QoL), measured through the Obesity-Related Well-being Questionnaire (ORWELL 97).

Step	Variables		B [95% CI]	*SE*	*Β*	*t*	*p*
**Step 1**	**(Constant)**		61.34 [17.65, 105.04]	21.97	-	2.79	0.007
	**PBI**	Care (mother)	0.11 [−0.38, 0.60]	0.25	0.05	0.43	0.665
		Overprotection (mother)	0.01 [−0.52, 0.54]	0.27	0.00	0.02	0.983
		Care (father)	−0.26 [−0.58, 0.07]	0.16	−0.16	−1.56	0.122
		Overprotection (father)	−0.45 [−1.01, 0.10]	0.28	−0.18	−1.62	0.108
	**ASQ**	Confidence	−0.63 [−1.33, 0.08]	0.35	−0.20	−1.77	0.081
		Discomfort with closeness	0.03 [−0.47, 0.53]	0.25	0.01	0.11	0.910
		Relationships as secondary	−0.39 [−0.93, 0.15]	0.27	−0.17	−1.42	0.159
		Need for approval	−0.05 [−0.72, 0.62]	0.34	−0.02	−0.15	0.885
		Preoccupation with relationships	0.85 [0.24, 1.47]	0.31	0.35	2.75	0.007 **
	**BES**	Total score	0.44 [0.08, 0.80]	0.18	0.24	2.46	0.016 *
**Step 2**	**(Constant)**		60.91 [5.90, 115.92]	27.63	-	2.21	0.030
	**PBI**	Care (mother)	0.37 [−0.14, 0.87]	0.26	0.18	1.43	0.157
		Overprotection (mother)	0.15 [−0.37, 0.66]	0.26	0.07	0.57	0.572
		Care (father)	−0.18 [−0.54, 0.17]	0.18	−0.11	−1.03	0.305
		Overprotection (father)	−0.40 [−0.95, 0.15]	0.28	−0.16	−1.44	0.153
	**ASQ**	Confidence	−0.75 [−1.45, −0.05]	0.35	−0.24	−2.12	0.037 *
		Discomfort with closeness	0.16 [−0.35, 0.66]	0.25	0.07	0.62	0.536
		Relationships as secondary	−0.36 [−0.92, 0.20]	0.28	−0.16	−1.28	0.204
		Need for approval	0.05 [−0.61, 0.71]	0.33	0.02	0.16	0.876
		Preoccupation with relationships	0.83 [0.21, 1.44]	0.31	0.34	2.69	0.009 **
	**BES**	Total score	0.57 [0.21, 0.94]	0.18	0.32	3.11	0.003 **
	**DA variables**	Age	0.01 [−0.23, 0.26]	0.12	0.01	0.11	0.916
		BMI	−0.32 [−1.08, 0.44]	0.38	−0.08	−0.84	0.402
	**Clinical variables**	Hypertension	−5.55 [−11.01, −0.08]	2.74	−0.20	−2.02	0.047 *
		Dyslipidemia	3.54 [−2.22, 9.30]	2.89	0.12	1.22	0.225
		Diabetes type 2	−7.40 [−13.03, −1.77]	2.83	−0.25	−2.62	0.011 *

*Legend.* PBI, Parental Bonding Instrument, ASQ, Attachment Style Questionnaire; BES, Binge Eating Scale; DA, Demographic and Anthropometric. *Notes. p* < 0.05 (*); *p* < 0.01 (**). Outcome variable: Quality of Life (QoL). Variables entered in Step 1: psychological dimensions (i.e., PBI, ASQ, and BES); Variables added in Step 2: covariates (i.e., demographic and anthropometric variables and clinical variables). Step 1: Adjusted R^2^ = 0.236; F (10, 92) = 3.839; Step 2: Adjusted R^2^ = 0.311; F (15, 92) = 3.769.

## Data Availability

Data and materials are available upon reasonable request.

## Supplementary Materials

The following supporting information can be downloaded at: https://www.mdpi.com/article/10.3390/bs15030305/s1, Table S1: Marital status and type of occupation prevalence for the overweight/obesity group (n = 96).
