# Supplementary material for: Exploring Attachment Dimensions in Individuals with Overweight or Obesity
Source: Behav Sci (Basel). 2025 Mar 4;15(3):305. doi: 10.3390/bs15030305 (PMC11939372; doi:10.3390/bs15030305)
Supplement: Supplementary file 1 [file behavsci-15-00305-s001.zip › behavsci-3479606-supplementary.pdf]

Table S1. Marital status and type of occupation prevalence for the overweight/obesity group (n = 96).

Variable	Answer	n, %
**Marital Status**	Married	59 (61.5%)
	In a committed relationship	14 (14.6%)
	Widowed	9 (9.4%)
	Separated or divorced	8 (8.3%)
	Single	3 (3.1%)
	Missing	3 (3.1%)
**Occupation**	Full-time employment	38 (39.6%)
	Part-time employment	3 (3.1%)
	Homemaker	10 (10.4%)
	Unemployed	2 (2.1%)
	Student	1 (1%)
	Retiree	8 (8.3%)
	Other	3 (3.1%)
	Missing	31 (32.3%)
